# Use of a Dual Reporter Plasmid to Demonstrate Bactofection with an Attenuated AroA^-^ Derivative of *Pasteurella multocida* B:2

**DOI:** 10.1371/journal.pone.0071524

**Published:** 2013-08-12

**Authors:** Sarah Othman, Andrew J. Roe, Roger Parton, John G. Coote

**Affiliations:** Institute of Infection, Immunity and Inflammation, College of Medical, Veterinary and Life Sciences, University of Glasgow, Glasgow, United Kingdom; Queen’s University, Canada

## Abstract

A reporter plasmid pSRG has been developed which expresses red fluorescent protein (RFP) from a constitutive prokaryotic promoter within *Pasteurella multocida* B:2 and green fluorescent protein (GFP) from a constitutive eukaryotic promoter within mammalian cells. This construct has been used to determine the location and viability of the bacteria when moving from the extracellular environment into the intracellular compartment of mammalian cells. Invasion assays with embryonic bovine lung (EBL) cells and an attenuated AroA^-^ derivative of *Pasteurella multocida* B:2 (strain JRMT12), harbouring the plasmid pSRG, showed that RFP-expressing bacteria could be detected intracellularly at 3 h post-invasion. At this stage, some EBL cells harbouring RFP-expressing bacteria were observed to express GFP simultaneously, indicating release of the plasmid into the intracellular environment. At 5 h post-invasion, more EBL cells were expressing GFP, while still harbouring RFP-expressing bacteria. Concurrently, some EBL cells were shown to express only GFP, indicating loss of viable bacteria within these cells. These experiments proved the functionality of the pSRG dual reporter system and the potential of *P. multocida* B:2 JRMT12 for bactofection and delivery of a DNA vaccine.

## Introduction

The numerous serotypes of *Pasteurella multocida* are associated with a variety of disease syndromes in a wide range of agricultural, domestic and feral animal species [1]. *P. multocida* serotypes B:2 and E:2 are associated with haemorrhagic septicaemia (HS) of cattle, water buffaloes and occasionally other species, resulting in major economic losses, mainly in South East Asia [2], [3]. Several types of killed, whole-cell vaccines have been used for prevention and control of the disease including alum-precipitated vaccine and oil-adjuvant vaccine but, in general, immunity is short-lived. Countries where the disease is endemic resort to routine prophylactic vaccination. A live-attenuated vaccine for HS might be a better alternative vaccine as, by mimicking the ability of the pathogen to infect the host, but without causing disease, a more prolonged protective immune response may be induced. A live-attenuated vaccine developed by Tabatabaei *et al.* [4], by mutating a housekeeping gene *aroA*, proved to be highly attenuated yet protective in mice and in cattle [4]–[6]. In later work, Othman *et al.* [7] reported on the ability of the live vaccine strain to adhere to and to invade embryonic bovine lung (EBL) cells with an efficiency similar to the wild-type strain. The live vaccine strain was also able to survive intracellularly in the EBL cells for at least 7 hours.

The long term aim of the present work is to exploit the capacity of the live *P. multocida aroA* vaccine strain to invade mammalian cells in order to deliver plasmid DNA encoding a protective antigen for another disease of the target animal, thus providing heterologous protection against both HS and a second disease. Bactofection, bacteria-mediated transfer of expressible DNA, is a promising approach to express plasmid-encoded heterologous proteins in different cell types, including phagocytic and non-phagocytic mammalian cells. Various studies have demonstrated bactofection with attenuated strains of invasive bacteria. For example, vaccination of mice with attenuated *Salmonella typhimurium* transformed with plasmid DNA encoding listeriolysin (a virulence factor of *Listeria monocytogenes*) induced specific antibody as well as T-cell responses to listeriolysin [8]. In a separate study, fluorescent dendritic cells (DC) were demonstrated after oral administration of *S. typhimurium* harboring plasmid DNA encoding green fluorescent protein (GFP). These data provided evidence that this delivery system could target relevant immune cells, leading to efficient induction of an immune response [9]. Mice vaccinated with an attenuated *Shigella* vaccine harbouring measles virus genes induced a vigorous measles virus antigen-specific response [10]. An attenuated *L. monocytogenes* strain has also been used for delivery of eukaryotic expression vectors to the cytoplasm of murine macrophage cell lines [11]. This technology has also been demonstrated with a plant pathogen, *Agrobacterium tumefaciens,* which contains a virulence plasmid that promotes transfer of an expression cassette from the plasmid into the plant. Although wounded plants are its natural targets, plasmid transfer by *A. tumefaciens* has also been demonstrated to yeast and fungi. *In vitro*, it can also target human cell lines such as HeLa cells [12]. These reports clearly demonstrate bacterial versatility as delivery vectors. As this approach has not previously been used with *Pasteurella,* we have developed a bactofection model system for use with this organism that employs a dual-expression plasmid for protein expression in both prokaryotic and eukaryotic cells in order to understand the fate of the plasmid DNA carrying antigenic genes after delivery by the live-vaccine strain *in vivo*.

## Materials and Methods

### Bacterial Strains and Growth Conditions

Bacterial strains used in this study were: *P. multocida* B:2 JRMT12, an *aroA* deletion mutant of *P. multocida* strain 85020 [4] and *E. coli* XL-1BLUE (Amersham Biosciences). *P. multocida* was grown on Brain Heart Infusion (BHI) agar or in BHI broth (Difco) media and *E. coli* was grown on Luria-Bertani (LB) agar or in LB broth (Difco) media at 37°C and shaken at 180 rpm.

### Plasmids

Plasmid pMK-Express [13] is a broad-host range plasmid that can propagate in HAP (*Haemophilus, Actinobacillus* & *Pasteurella*) bacteria. An *A. pleuropneumoniae* promoter, *^P^*sod-C, was incorporated into plasmid pMK-Express upstream of the *gfpmut3* gene coding for GFP. This constitutive promoter was able to express the GFP protein in *Pasteurella* [13]. Plasmid pDsRed-Monomer (Clontech) encodes red fluorescent protein (RFP) from the DsRed fragment. Plasmid pEGFP-N1™ (Clontech) encodes green fluorescent protein (GFP) from a constitutive eukaryotic promoter *^P^*CMV_IE_ and is able to replicate in *E. coli* and eukaryotic cells.

### General Molecular Biology Techniques

Plasmid extractions were performed using Qiaprep spin columns (Qiagen). DNA concentrations were measured using a NanoDrop ND-1000 UV-visible spectrophotometer (NanoDrop Technologies). Unless otherwise stated, restriction enzymes were obtained from New England Biolabs and used according to the manufacturer’s protocol. PCR was performed according to standard procedures [13], [14] using *Taq* DNA polymerase (NEB,UK). RED primers with internal *Pci*I sites (bold fonts) (RED Forward: 5′- CT**ACATGT**CCGCGCCAACCG −3′, RED Reverse: 5′- GC**ACATGT**TCTACTGGGAG -3′) were used to amplify the *P_sod_*_C_-DsRed fragment from plasmid pMK-RED. A MegaBACE1000 (96 capillary) sequencer, which used Big Dye (Applied Biosystems) and ET-Dye Terminator (Amersham Bioscience) chemistries, was employed for DNA sequencing. The sequencing was done by The Sequencing Unit, University of Dundee, Scotland.

### Plasmid Transformation

An overnight culture of *P. multocida* grown in BHI broth was diluted 1 in 100 into 500 ml of pre-warmed BHI broth and grown at 37°C to an optical density at 600 nm (OD_600nm_) of 0.5–0.7 measured using a cell density meter (WPA CO8000, Biochrom Ltd.). The cells were harvested by centrifugation and then made electrocompetent by concentrating 100-fold and washing three times with ice-cold 10% (v/v) glycerol before suspension in ice-cold 10% (v/v) glycerol. Electrocompetent cells (50 µl) plus 10–200 ng of PCR product, plasmid or ligation mixture were used. After mixing, electroporation was done with a Bio-Rad pulse controller (MicroPulser Electroporator, Bio-Rad) set at 2.5 kV (field strength 12.5 kV/cm^2^). Shocked cells were added to 1 ml of BHI and incubated at 37°C for 4 h. Aliquots of 50–100 µl of transformed cells were then spread onto selective plates containing appropriate antibiotics and the plates were incubated at 37°C to obtain transformant colonies.

For *E. coli,* plasmid DNA was transformed into *E. coli* XL-1BLUE using the heat shock protocol specified by the manufacturer (Amersham Biosciences).

### Preparation of Embryonic Bovine Lung (EBL) Cells

EBL cells (German Collection of Microorganisms and Cell Cultures, no. ACC192) were grown as a monolayer in Minimal Essential Medium (MEM 2279, Sigma) containing streptomycin (100 mg/ml), penicillin (100 U/ml) and 15% (v/v) foetal calf serum (FCS, Sigma) in a humidified atmosphere of 5% (v/v) CO_2_ and 95% (v/v) air at 37°C. Cells taken from liquid nitrogen stock were cultured for two days before seeding into a 24-well tissue-culture plate and incubated overnight.

### EBL Cell Viability Assessment

The trypan blue exclusion method was used. A mixture containing 10 µl of trypan blue (0.4% w/v) (Sigma) and 10 µl of the cell suspension was placed in an improved Neubauer (1/400 mm^2^×0.1 mm depth) chamber and viewed with a light microscope and a ×10 objective lens. Cells stained blue were counted as dead whereas clear cells were counted as viable.

### *P. multocida* Invasion of EBL Cells

This was done as previously described (Othman *et al.,* 2012) (N.B. ref [7]). Briefly, bacteria were harvested from 18-h cultures in BHI broth, washed twice in phosphate-buffered saline (PBS) and suspended in MEM without antibiotics. The bacterial suspension was adjusted to an OD_600nm_ corresponding to approximately 1×10^7^ colony forming units (CFU)/ml to allow a final multiplicity of infection (MOI) of 100 bacteria/mammalian cell. Previous work had shown this to be the optimum MOI in our system (7). The concentration of viable EBL cells in one well of a 24-well tissue culture plate was assessed by trypan blue and bacterial suspension was then added at the appropriate concentration to each well containing EBL monolayer. The plate was centrifuged at 1200×g for 5 min in a Heraeus 3 S-R centrifuge to encourage close contact between the EBL and bacterial cells. After incubation in a CO_2_ incubator at 37°C for 2 h, the wells were washed gently with PBS to remove the unattached bacteria. The washing step was repeated twice. One millilitre of MEM with antibiotics [polymyxin (50 µg/ml) and gentamicin (50 µg/ml)] was added to each well and the plate was incubated for 1 h at 37°C in 5% (v/v) CO_2_ to kill any remaining extracellular bacteria. The wells were then washed twice with PBS to remove the antibiotics before further manipulation.

### Assessment of RFP and GFP Expression in Bacteria

This was monitored using a fluorimeter (FLUOstar OPTIMA, BMG Labtech). BHI broth (100 ml), supplemented with appropriate antibiotics at a final concentration of 50 µg/ml, was inoculated with 1 ml of an overnight culture of *P. multocida* B:2 or *E. coli* XL-1BLUE and shaken at 180 rpm, 37°C. At hourly intervals, 1 ml samples of culture were taken and the OD_600nm_ was measured. The suspension was then centrifuged at 13 000 x g for 1 min and the pellet resuspended in 1 ml of PBS. The OD_600nm_ was measured again with PBS as standard and 200 µl aliquots placed in wells of a 96-well flat-bottom dish (Corning, USA) in triplicate. The fluorescence intensity (FI) at 550 nm for GFP and 590 nm for RFP was measured in the fluorimeter with a fluorescent light filter and excitation at 490 nm and 520 nm, respectively. All samples were compared with bacteria without plasmids to eliminate background fluorescence. Samples were taken at hourly intervals until the OD_600nm_ reached stationary phase (∼2.0). Control experiments showed negligible expression of GFP in *P. multocida* JRMT12 throughout the growth cycle, indicating that the *^P^*CMV_IE_ promoter was not able to drive transcription of GFP in this prokaryotic background.

### Fluorescence Imaging

#### Fluorescent staining of EBL cells

EBL cells were treated according to the invasion assay above. The only difference for fluorescence microscopy (FM) slide preparation was that the EBL cells were seeded in a chamber-slide (NUNC) rather than a 24-well plate, so that cells could be fixed directly to the slide after removing the well from the chamber slide.

After antibiotic treatment to eliminate extracellular and adherent bacteria, the monolayer was washed twice with PBS. CellMask plasma membrane stain (GE Healthcare) (5 µg/ml) (diluted according to manufacturer’s instructions) was added at 200 µl per well. The chamber-slide was then incubated for 7 min at 37°C in an atmosphere supplemented with 5% (v/v) CO_2_. The staining solution was removed from the well and the cells washed three times with PBS. Pre-warmed paraformaldehyde (PFA) 200 µl at 4% (v/v) was added to each well and slides incubated for 30 min at 37°C in an atmosphere supplemented with 5% (v/v) CO_2_. PFA was then removed and cells were washed three times with PBS. The wells were then removed from the chamber-slide and the slide was left to dry.

Fluorescence mounting medium (DAKO) was used to cover the fixation area on the chamber-slide. A cover slip was then gently applied on top of the medium to avoid air bubble formation. Any gap between the cover slip and the chamber-slide was then sealed-off using nail varnish to prevent air pockets from forming. The slide was left for 10 min to allow the mounting medium to set. It was then cleaned and labelled before storage in the dark.

### Fluorescent Staining of Bacteria

Bacteria were grown in BHI broth with vigorous shaking at 37°C until an OD_600nm_ of 0.3–0.5 was obtained. One millilitre of bacterial suspension was taken and centrifuged at 13 000×g for 1 minute. The pellet was resuspended in 1 ml of PBS and the OD_600nm_ was measured. An aliquot of 200 µl was transferred from the PBS suspension into a flat-bottom 96-well plate. All samples were done in triplicate. Fluorescence intensity (FI) of RFP was then measured.

A vial of Cye5Dye (Amersham Bioscience) was mixed with 100 µl of PBS according to the manufacturer’s instructions and the dye was then mixed with bacterial suspension at a ratio of 1∶20 (25 µl of dye to 500 µl of bacterial suspension). The mixture was then incubated in the dark at room temperature for 1 h to allow staining of bacterial free-peptide groups on the outer membrane. After incubation, the suspension was pelleted at 13 000×g for 1 min and washed with 1 ml of PBS before being re-centrifuged. The washing step was repeated until the suspension was no longer coloured.

For fixation, an aliquot of the suspension was mixed (1∶1) with 4% (v/v) PFA. About 20 µl of the fixation mixture was spotted onto a clean spot-slide which was then left at room temperature to dry. Fluorescence mounting medium (DAKO) was used to cover the fixation area on the spot-slide and the slides prepared for storage as described.

### Fluorescence Microscopy (FM)

Fluorescence imaging was done with a Zeiss AxioImager M1 upright fluorescence microscope (Carl Zeiss) connected to a Hamamatsu Orca 03 and QIClick digital charge-coupled device (CCD) camera. The system allows visualization of fluorescence at high spatial resolution (1200×1300 pixels) and high bit depth (12-bit gray scale). Acquisition, visualization, measurement and restoration of the images were then processed with Volocity software (Perkin Elmer).

## Results

### Construction of “traffic-light” Plasmid, pSRG

**Figure 1:**
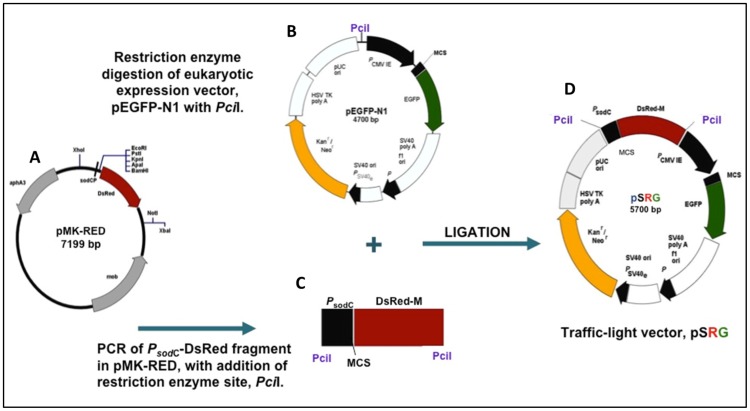
Construction of the “traffic light” reporter plasmid, pSRG. (A) Plasmid pMK-RED was constructed from plasmid pMK-Express [13] containing the DsRed.M1 gene from plasmid pDsRed-Monomer (Clontech) inserted between the *Bam*HI and *Not*I sites in place of the *gfpmut3* gene. (B) Eukaryotic expression plasmid pEGFP-N1™ (Clontech) was digested with *Pci*I. (C) Amplification of the *^P^*sodC-DsRed fragment with RED primers containing *Pci*I sites. (D) Plasmid pSRG with two expression systems, a red reporter system that functions in prokaryotic cells driven by the *sodC* promoter and a green reporter system that functions in eukaryotic cells driven by the *^P^*CMV_IE_ promoter.

Fig. 1 shows the cloning strategy for the construction of pSRG. First, plasmid pMK-RED was generated. It was derived from plasmid pMK-Express [13] and contained the DsRed fragment from pDsRed-Monomer (Clontech). The *gfpmut3* gene from pMK-Express was removed by digestion with *BamH*I and *Not*I and replaced by the DsRed fragment cut from pDsRed-monomer to produce pMK-RED (Fig. 1A). Plasmid pMK-RED was then used as a template for PCR with RED primers to amplify the *P_sod_*_C_-DsRed fragment with *Pci*I sites at each end. This amplimer was cut with *Pci*I and cloned into the unique *Pci*I site of pEGFP-N1 (Fig. 1B, C). Ligation of this fragment into pEGFP-N1 was performed at a ratio of 1∶2 (plasmid:fragment) to obtain plasmid pSRG (Fig. 1D). The ligation mixture was then transformed into *E. coli* XL-1BLUE. Plasmid pSRG was purified using Qiaprep Spin Miniprep Kit (Qiagen) according to manufacturer’s instruction and confirmed by restriction enzymes digestion.

After that, pSRG was electroporated into *P. multocida* B:2 JRMT12 and positive transformants were selected on BHI agar supplemented with kanamycin (50 µg/ml). A strain harbouring pSRG was assessed for plasmid stability after repeated subculture. *P. multocida* B:2 JRMT12 pSRG was plated on BHI agar with and without kanamycin (50 µg/ml). After incubation for 24 h, selected colonies from both plates were streaked to fresh plates. After a further 24 h, a dozen colonies were picked from both plates and inoculated into BHI broth with or without kanamycin (50 µg/ml) respectively. After 24 h, plasmids were purified and analysed by restriction digestion with *Pci*I for confirmation of their identity. Strains were subcultured daily into fresh broth medium for 14 days and plasmids from each culture were purified and analysed every alternate day. After 14 days, *P. multocida* B:2 JRMT12 pSRG cultured without antibiotic was found to be still harbouring the plasmid in each of the 12 clones subcultured. Thus, pSRG was stably maintained in *P. multocida* B:2 JRMT12 in the absence of antibiotic.

### Expression of RFP from pSRG in *P. multocida* B:2 JRMT12

**Figure 2:**
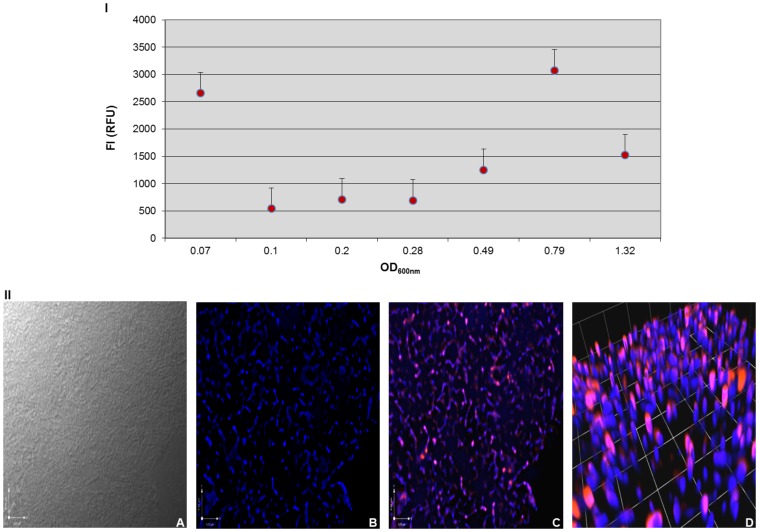
(I) Assessment of RFP expression by *P. multocida* B:2 JRMT12 pSRG during growth. *P. multocida* B:2 JRMT12 without plasmid acted as a control for background fluorescence. Fluorescence was plotted against OD_600nm_ using Microsoft Excel software and data were corrected for background fluorescence. Experiments were performed in triplicate and the error bars indicate standard deviations of the means. (**II**) **Visualization of** ***P. multocida*** **B:2 JRMT12 pSRG via fluorescence microscopy.** Images of *P. multocida* B:2 JRMT12 pSRG were taken of the same field under three different light paths; **A**: DIC (white) light, **B**: filtered fluorescent light (Exc. = 649 nm, Ems. = 670 nm) (blue) to visualize the counterstain, Cy5 Dye (Amersham Bioscience), **C**: two filtered lights; filtered fluorescent light (Exc. = 557 nm, Ems. = 585 nm) (red) to visualize bacteria expressing RFP from plasmid pSRG and filtered fluorescent light (Exc. = 649 nm, Ems. = 670 nm) (blue) to visualize the counterstain, Cy5 Dye in order to show that RFP expression was within the bacteria, **D**: 3D imaging of the 2D image from C using Volocity software.

Figure 2 (I) shows the fluorescence intensity (FI) in relative fluorescence units (RFU) of *P. multocida* B:2 JRMT12 pSRG plotted against bacterial density (OD_600nm_). Immediately after inoculation into BHI broth, at OD_600nm_ 0.07, the strain was clearly expressing RFP (FI >2500 RFU). This fluorescence decreased initially but then increased with increasing bacterial density, to >3000 RFU when the OD_600nm_ had reached 0.79. After that, the RFP expression was seen to decline. The results indicated that the RFP expression from this strain peaked in the late exponential phase of growth.

### Visualization of RFP Expression from *P. multocida* B:2 JRMT12 pSRG via Fluorescence Microscopy (FM)

The bacteria were cultured as described before, and sampling was done between OD_600nm_ 0.7 and 0.8 in order to capture the bacteria during maximum RFP expression. After confirming RFP expression, in the fluorimeter, bacteria were prepared for microscopy.

In Figure 2 (II), a set of images was captured from the spot slide prepared with *P. multocida* B:2 JRMT12 pSRG taken at OD_600nm_ 0.79. Images were taken of the same field using the 100× objective lens under three different light paths to ease visualization. Image **A**, taken under DIC (Differential Interference Contrast) (white) light, showed a heavy cluster of bacteria. When the same field of the slide was captured in **B,** where the image was taken under filtered fluorescent light (Excitation = 649 nm, Emission = 670 nm) (blue) to visualize the counterstain Cy5 Dye, the bacteria were more defined. This image was taken at 0.6 µm splicing through the slide in order to view a single layer of the bacteria. The slide was set to be sectioned optically at 0.3 µm intervals from top to bottom; an image was captured at every 0.3 µm depth at a total section stacking of 3.0 µm. The Volocity software was then utilized to deconvolve images captured by reversing the optical distortion to produce a sharper and clearer image. This technique also eliminated background light diffraction. Image **C** was taken under filtered fluorescent light (Exc. = 557 nm, Ems. = 585 nm) (red) to visualize bacteria expressing RFP from plasmid pSRG and under filtered fluorescent light (Exc. = 649 nm, Ems. = 670 nm) (blue) to show the counterstain in order to localize the RFP expression in the bacteria. The image showed purplish pink coccobacilli of *P. multocida* B:2 JRMT12 pSRG expressing RFP. Image **D** shows a 3-dimensional (3D) image of image **C** to generate a 3D model of the RFP-expressing bacteria. The vertical elongated shape of the bacteria is presumed to be an artefact of iterative restoration after image manipulation using Volocity software.

### Intracellular Trafficking of *P. multocida* B:2 JRMT12 pSRG within EBL Cells

EBL cells and bacteria were prepared as above but with the modification that the bacterium, *P. multocida* B:2 JRMT12 pSRG, was grown to OD_600nm_ 0.79 (at maximum RFP expression) before use in the invasion assay. Slides were prepared for FM and viewed under the x100 objective lens of the Zeiss AxioImager M1 microscope (Carl Zeiss).

**Figure 3:**
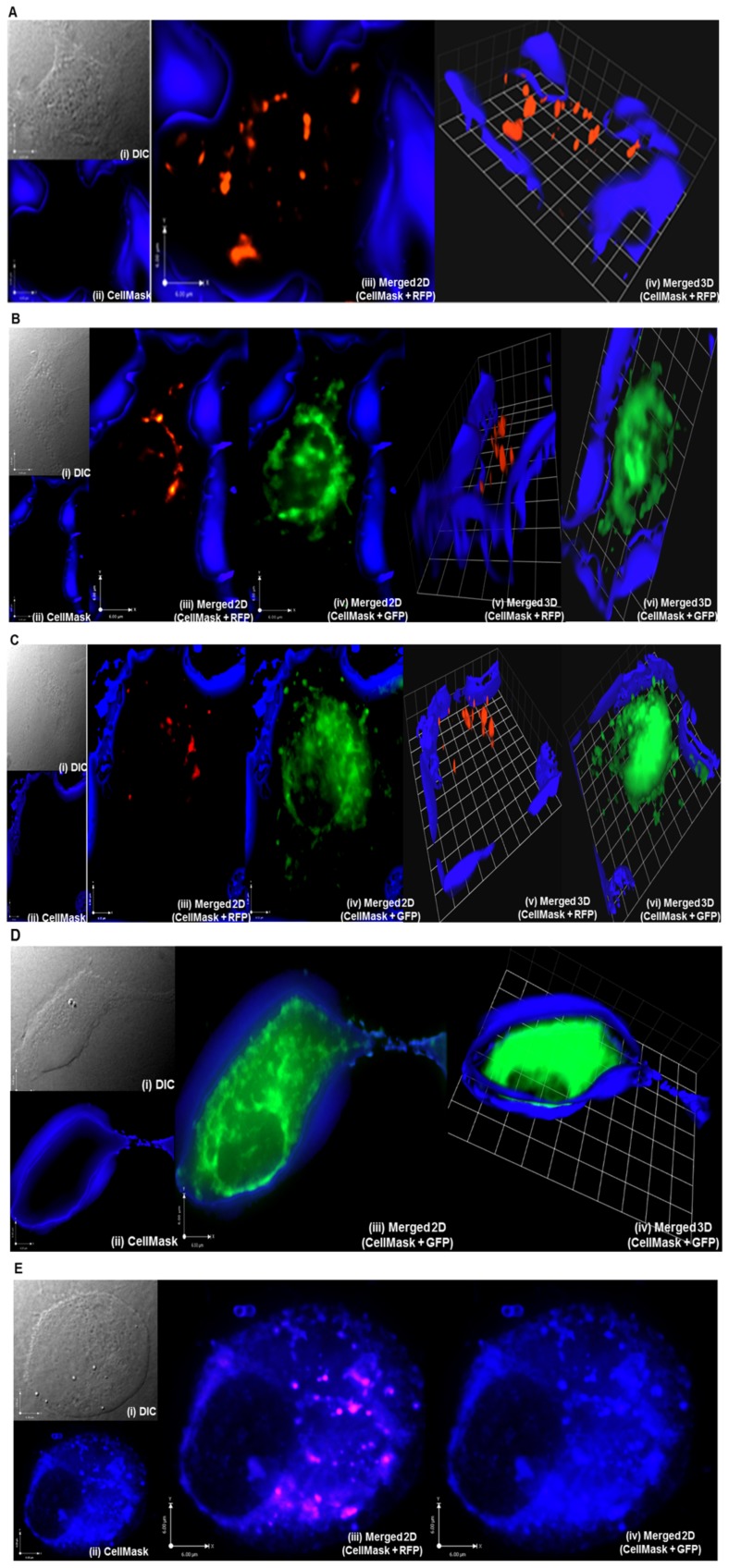
Localization of prokaryotic and eukaryotic protein expression. The images show EBL cells after invasion with *P. multocida* B:2 JRMT12 pSRG. Images were captured at 3 h (**A** and **B**) and 5 h (**C** and **D**) post-invasion. In **A**, an EBL cell was viewed (**i**) under DIC (white) light, (**ii**) by counterstaining with CellMask™ plasma membrane stain (GE Healthcare) (blue), (iii) merged 2D images of (ii) and image captured under filtered fluorescent light (Exc. = 557 nm, Ems. = 585 nm) (red) to visualize internalized RFP-expressing bacteria and (iv) a 3D image of (iii). In B and C, an EBL cell was viewed (i) under DIC (white) light, (ii) by counterstaining with CellMask™ plasma membrane stain (GE Healthcare) (blue), (**iii**) merged 2D images of (ii) and image captured under filtered fluorescent light (Exc. = 557 nm, Ems. = 585 nm) (red), (**iv**) merged 2D images of (ii) and image captured under filtered fluorescent light (Exc. = 488 nm, Ems. = 507 nm) (green) to visualize expression of GFP by the mammalian cells, (v) a 3D image of (iii) and (**vi**) a 3D image of (iv). In **D**, an EBL cell was viewed (**i**) under DIC (white) light, (**ii**) by counterstaining with CellMask™ plasma membrane stain (GE Healthcare) (blue), (**iii**) merged 2D images of (ii) and image captured under filtered fluorescent light (Exc. = 488 nm, Ems. = 507 nm) (green) and (**iv**) a 3D image of (iii). The merged 3D images were included to demonstrate the localization of either RFP or GFP expression. Fluorescence emitted by EBL cells infected with JRMT12 strain harbouring pMK-RED (**E**) with the same microscope settings, was used as a negative control. Each cell was viewed (i) under DIC (white) light, (**ii**) by counterstaining with CellMask™ plasma membrane stain (GE Healthcare) (blue), (**iii**) merged 2D images of (ii) and image captured under filtered fluorescent light (Exc. = 557 nm, Ems. = 585 nm) (red) to visualize internalized RFP-expressing bacteria and (**iv**) merged 2D images of (ii) and image captured under filtered fluorescent light (Exc. = 488 nm, Ems. = 507 nm) (green).

Five sets of images are displayed in Figure 3. Sets A and B were captured from slides prepared after the standard invasion time of 3 h, while sets C and D were captured from slides prepared at 5 h post-invasion. All images in a set were captured in the same field of the slides but under different light paths. In set A, the first image (i) shows a single EBL cell taken under DIC (white) light. Under blue-filtered fluorescent light, the plasma membrane was stained blue (ii). An overlay of blue- and red-filtered fluorescent light showed the cell membrane as blue and the internalized *P. multocida* B:2 JRMT12 pSRG as red (iii). The 3D image (iv) displayed was generated from the 2D image using Volocity software. However, when the fluorescent light path was changed to green to visualize GFP, no green fluorescence was found to be expressed by this EBL cell (not shown). Hence, at 3 h post-invasion, internalized bacteria were found in this EBL cell but no expression of GFP was detected. This indicated that the bacteria were still intact and had not released plasmid into the intracellular environment of the EBL cell.

As in set A, images in set B were also captured at 3 h post-invasion. In this set, again a single EBL cell is clearly defined under the DIC (white) light (i) and filtered fluorescent blue light demonstrated the EBL cell membrane in blue when stained by the plasma membrane stain CellMask (ii). Under red and blue filtered fluorescent light, RFP expression (red) was visualised as in image A (iii). By switching the filtered fluorescent light path into green and blue, the EBL cell was seen to express GFP within the cell cytoplasm (iv). 3D images (v & vi) were then generated from the 2D images using the Volocity software. The data could be interpreted to indicate that, in this EBL cell, free plasmid was available for expression of GFP in the cytoplasm of the cell. The presence of fewer RFP-expressing bacteria within this cell might mean that others had been lysed so that they were no longer able to express RFP but had released plasmid into the cytoplasm to generate GFP. The obvious presence of bacteria at the periphery of the EBL cell, expressing RFP, might indicate that these bacteria had not yet been taken up by the cell but had nevertheless resisted washing and antibiotic treatment.

Seventy EBL cells were assessed for RFP or GFP expression from slides prepared at 3 h post-invasion. Of these cells, 39 (56%) were found not to be expressing GFP and no RFP-expressing bacteria were found intracellularly. The remaining 31 (44%) EBL cells were found to harbour a number of RFP-expressing *P. multocida* B:2 JRMT12 pSRG intracellularly and, of these, 12 (17% of total) EBL cells were already expressing GFP. No EBL cells expressing GFP without harbouring RFP-expressing bacteria were found at this stage.

Images in sets C and D were captured at 5 h post-invasion. In set C, under DIC (white) light, a single EBL cell on a monolayer was captured (i). When viewed under filtered fluorescent blue light, the labelled EBL membrane boundaries were visible as a blue fluorescence (ii). When viewed with red and blue filtered fluorescent light paths, the image showed some internalized bacteria (red) within membrane boundaries (blue) (iii). Green and blue fluorescent light paths showed green fluorescence in the EBL cell cytoplasm within membrane boundaries (blue) (iv). Both 2D images were then processed with Volocity software to generate their respective 3D images. In these, localization of RFP (v) and GFP (vi) expression was clearly displayed within the labelled (blue) membrane boundaries.

Set D images, captured at 5 h post-invasion, show a single EBL cell taken under DIC (white) light (i) and the same cell viewed under filtered fluorescent blue light revealed the labelled cell membrane (ii). Switching to both green and blue filtered fluorescent light showed expression of GFP by the EBL cell, displayed as green fluorescence within the cell membrane (blue) (iii). The 3D image (iv) was then generated from the 2D image using Volocity software. When the cell was viewed under red and blue filtered fluorescent light paths, the image showed only the EBL cell with the membrane boundaries outlined in blue fluorescence (image not shown). These data indicated that, at this stage, internalized RFP-expressing bacteria had been lysed so that they were no longer able to express RFP but had released the plasmid into the EBL cytoplasm for GFP expression controlled by the eukaryotic promoter.

As a negative control, EBL cells were infected with the JRMT12 strain harbouring pMK-RED (Figure 1), which expresses only RFP. This control was analysed using the same microscopy settings as for sets A–D. Panel E shows that internalised bacteria were expressing RFP while the infected EBL cells emitted no detectable green autofluorescence. This confirms the conclusion that fluorescence measured in the GFP channel as in panels B-D could only be attributed to expression from pSRG.

Sixty EBL cells were assessed from slides prepared at 5 h post-invasion. Of these, 28 (47%) were found not to be expressing GFP and no RFP-expressing bacteria were found intracellularly. Each of the remaining 32 (53%) EBL cells were found to be expressing GFP and of these, 12 (20% of total) cells were found to be still harbouring some RFP-expressing *P. multocida* B:2 JRMT12 pSRG intracellularly whereas the others expressed only GFP.

Images A (iii), B (iii) and C (iii) show the capacity of *P. multocida* B:2 JRMT12 pSRG, internalised within EBL cells, to express RFP from pSRG. RFP expression would have taken place within intact bacteria, driven by the *^P^*sod-C promoter. Images B (iv), C (iv) and D (iv) show the ability of EBL cells to express GFP from pSRG which must have been released from the internalized *P. multocida* B:2 JRMT12 pSRG in order for the eukaryotic promoter *^P^*CMV_ IE_ to drive expression of GFP within the EBL cell cytoplasm. The images also illustrate the variation in the timing of both RFP and GFP expression with one cell showing RFP expression as late as 5 h post-invasion (image C) and another cell expressing GFP as early as 3 h (images A and B). From observation of 70 EBL cells at 3 h post-invasion, three categories of EBL cells were found. Some were found without any bacteria internally (no RFP expression), some had internalized bacteria but the mammalian cells were not expressing GFP and some, the lowest percentage, harboured internalized viable *P. multocida* B:2 JRMT12 pSRG but at the same time expressed GFP. Observation of 60 cells at 5 h post-invasion, demonstrated three categories of EBL cells, some without any bacteria internally, some harbouring internalized viable *P. multocida* B:2 JRMT12 pSRG expressing RFP while at the same time expressing GFP, and some EBL cells without any RFP expression but which expressed GFP. The last category was not found at 3 h post-invasion and the second category of EBL cells found at 3 h post-invasion was not found at 5 h post-invasion. Taken together, these data present good evidence that *P. multocida* B:2 JRMT12 can carry plasmid pSRG into EBL cells and, while the bacteria remain viable, they will express RFP. Loss of RFP expression at 5 h post-invasion plus expression of GFP would indicate release of pSRG into the cytoplasm from the non-viable bacteria, where the plasmid was able to express GFP from the *^P^*CMV_IE_ promoter.

## Discussion

Bactofection (bacteria-mediated transfer of expressible DNA) can be applied for genetic vaccination, gene therapy and production of therapeutic proteins [15]. A development of the work described here would be to apply bactofection to genetic vaccination against cattle diseases. Hence, in this study, our aim was to visualize trafficking of a potential delivery plasmid from *P. multocida* B:2 into EBL cells using a plasmid able to propagate and express a reporter protein in *P. multocida* and also to express a reporter protein in mammalian cells. As outlined in Figure I, the “traffic-light” plasmid termed pSRG was designed with a promoter, *^P^*sod-C, active in *Pasteurella* bacteria upstream of a DsRed.M1 gene coding for RFP, together with the virally-derived promoter, *^P^*CMV_IE_, for expression in eukaryotes, upstream of the EGFP gene coding for GFP. pSRG, at 5700 bp, was successfully propagated and maintained stably in the *P. multocida* B:2 strain JRMT12 for at least 14 days without the presence of selective antibiotic. Assessment of RFP expression through the bacterial growth cycle showed that JRMT12, harbouring pSRG, expressed RFP maximally during the late exponential phase. Fluorescence microscopy showed *P. multocida* B:2 JRMT12 pSRG expressing RFP from individual bacteria.

An invasion assay with *P. multocida* B:2 JRMT12 pSRG was undertaken to demonstrate bacterial entry, plasmid release and reporter protein expression within EBL cells. At 3 h post-invasion, bacteria were seen intracellularly, along with RFP expression and in some cells green fluorescence was localised only in the cytoplasm and not in the nucleus. FM images suggested that, at 3 h post-invasion, RFP-expressing bacteria were found intracellulary in EBL cells. Some of these cells were expressing GFP indicating that bacterial lysis had occurred in some cases and plasmid had been released into the cytoplasm. Images captured at 5 h post-invasion showed that some 20% of EBL cells still contained viable *P. multocida* B:2 JRMT12 pSRG as demonstrated by RFP expression. This confirmed findings of Othman *et al* [7], on the intracellular survival of the live-vaccine strain, that viable intracellular bacteria could be detected from 3–7 h post-invasion although the number decreased with time. FM images at 5 h post-invasion showed some EBL cells containing viable *P. multocida* B:2 JRMT12 pSRG were also expressing GFP, an indicator of bacterial lysis and the presence of pSRG in the EBL cell cytoplasm. The 3D images provided a better visualization of the model system to demonstrate localization of expression of each fluorescent protein. Other EBL cells captured at 5 h post-invasion were negative for RFP expression yet were expressing GFP. This suggested that all the internalised bacteria had been lysed and free plasmid DNA had been delivered outside of the phagocytic vacuoles. The 3D images showed localization of eukaryotic GFP expression in the cytosol of the EBL cell.

The high level of bacterial invasion (10–47%) found in this work and a previous study (7) indicates the potential of the system as a delivery method for foreign antigens. However, differences in behaviour are observed among attenuated bacterial strains in the hostile environment inside mammalian cells. Bacteria such as *S. flexneri, L. monocytogenes* or *E. coli,* carrying the virulence plasmid of *S. flexneri*, invade their host cells and then escape from a vacuole into the cytosol. Once in the cytosol they die, for example because they are auxotrophic, conditionally express autolysins or are killed by the addition of antibiotics to the cultures. This results in lysis of the bacteria and release of the expression plasmids. Some of these plasmids find their way into the nucleus where the open reading frames are expressed under the control of the eukaryotic transcription elements [16]–[19]. Bacteria such as *S. typhimurium*, *S. typhi* or *E. coli* expressing the invasion gene, *inv,* of *Y. pseudotuberculosis,* invade their host cells and remain in the vacuole. There they die, for example due to metabolic attenuation, and release their expression plasmids. By an unknown mechanism, these plasmids cross the vesicular membrane and reach the cell nucleus of the host cells where they are expressed [16], [20], [21]. Our previous work showed by transmission electron microscopy that invasive *aroA P. multocida* B:2 were confined to the vacuoles of EBL cells and some internalised bacteria appeared to be ruptured and in the process of degradation (7). Thus, it can be assumed that the attenuated *aroA* mutant dies through metabolic attenuation and then releases pSRG which must transfer from the vacuoles into the cytoplasm and then be delivered to the nuclear compartment, by an unknown mechanism. The above results have indicated that it is possible to obtain efficient bactofection using *P. multocida* B:2. However, in future work, more evidence of DNA transfer could perhaps be obtained by demonstrating splicing of the plasmid-encoded eukaryotic mRNA or expression in the presence of antibiotics that block prokaryotic protein synthesis.

**Figure 4:**
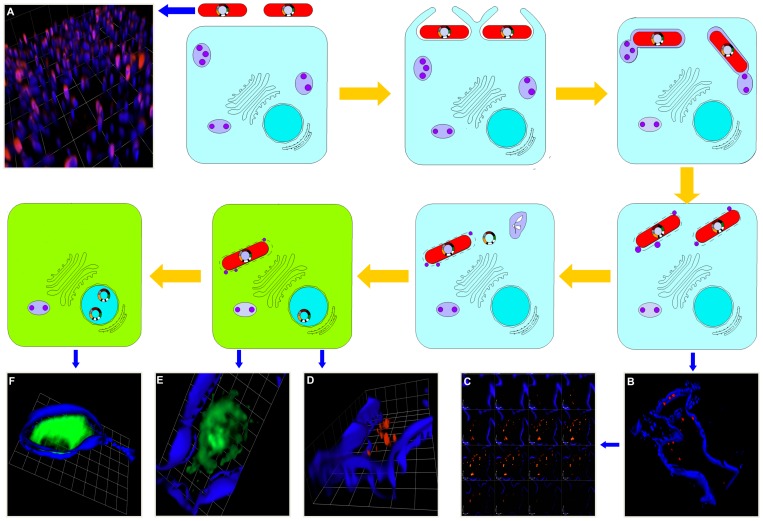
Bactofection pathway for DNA vaccine delivery. Yellow arrows indicate a schematic pathway for the bacterial plasmid delivery process in EBL cells. After internalization of the bacteria and phagolysosome fusion, the escape of the plasmid DNA (pSRG) from the vacuole into the cytosol after bacterial degradation results in the transfer of the pSRG to the intracellular environment of the EBL cell. Plasmid DNA then localises in the nucleus (blue circle) to allow expression of the GFP gene by the eukaryotic promoter, which is followed by GFP formation in the cytosol (green colouration in cytoplasm). Blue arrows point to microscopic images taken at different stages of the invasion assay; (A) *P. multocida* B:2 JRMT12 pSRG expressing RFP. Bacteria counterstained with Cy5 Dye (blue). (B) EBL cell at 3h post-invasion showing intracellular *P. multocida* B:2 JRMT12 pSRG expressing RFP. The EBL plasma membrane is counterstained blue. (C) Optical sectioning of the EBL cell in B at 0.3 µm intervals from top to bottom, confirmed the intracellular location of the RFP-expressing bacteria. (D) EBL cell in B at 3h post-invasion showing intracellular *P. multocida* B:2 JRMT12 pSRG expressing RFP. (E) The same EBL cell in D expressing GFP from intracellular plasmid DNA. (F) EBL cell expressing GFP at 5h post-invasion with *P. multocida* B:2 JRMT12 pSRG. Magnification x100; counterstaining with Cy5 Dye (blue) for bacteria and CellMask (blue) for EBL cells.

Figure 4 is a schematic diagram to model the bactofection pathway of *P. multocida* B:2 JRMT12 pSRG after infection of EBL cells and also incorporates some of the FM images obtained. In Figure 4A, *P*. *multocida* B:2 JRMT12 pSRG is shown expressing RFP before invading the EBL cells. At 3 h post-invasion, bacterial RFP expression was seen intracellularly (Figures 4B & 4C). Some infected cells harbouring viable *P. multocida* B:2 JRMT12 pSRG expressing RFP also expressed GFP (Figures 4D & 4E). This suggested that there was death of the bacteria from an early stage of infection. At 5 h post-invasion, the majority of the GFP-expressing cells were *Pasteurella*-negative (Figure 4F). In conclusion, our “traffic-light” plasmid pSRG, encoding dual promoters downstream of respective fluorescent reporters, represents a convenient and useful tool for analysing the different steps of the *P. multocida* B:2 infectious process by FM. Together with the live-attenuated *P. multocida* B:2 strain JRMT12, it has the potential to act as a vaccine, not only against HS, but also to provide immunity to other diseases, by replacing the eukaryotic GFP reporter gene with genes for protective antigens from other disease-causing organisms. In this regard, it will be of considerable value in future work to evaluate the persistence of GFP expression in EBL cells and, more importantly, to assess the persistence of antigen expression *in vivo.* In addition, our understanding of *Pasteurella* infection could be broadened by live-cell imaging for better visualization during time-course experiments and by flow-cytometry to further evaluate how bacterial dosage relates to plasmid delivery and protein expression.
